# Surrogate phenotype definition for alcohol use disorders: a genome-wide search for linkage and association

**DOI:** 10.1186/1471-2156-6-S1-S55

**Published:** 2005-12-30

**Authors:** Albert Rosenberger, Nico Janicke, Karola Köhler, Katrin Korb, Bettina Kulle, Heike Bickeböller

**Affiliations:** 1 Department of Genetic Epidemiology, Georg-August University Göttingen, Humboldtallee 32, 37073 Göttingen, Germany

## Abstract

For the identification of susceptibility loci in complex diseases the choice of the target phenotype is very important. We compared results of genome-wide searches for linkage or for association related to three phenotypes for alcohol use disorder. These are a behavioral score BQ, based on a 12-item questionnaire about drinking behavior and the subject's report of drinking-related health problems, and ERP pattern and ERP magnitude, both derived from the eyes closed resting ERP measures to quantify brain activity. Overall, we were able to identify 11 candidate regions for linkage. Only two regions were found to be related to both BQ and one of the ERP phenotypes. The genome-wide search for association using single-nucleotide polymorphisms did not yield interesting leads.

## Background

For the identification of susceptibility loci in complex diseases, genome-wide searches are a first step. Study design issues such as sample structure and marker choice play a role. However, of fundamental importance is a precise, homogeneous, and insightful phenotype definition. The right hunch might be the key to a particular disease pathway. The Genetic Analysis Workshop 14 (GAW14) family data on alcohol use disorders (short: alcoholism) provide the possibility to compose new phenotypes for alcoholism. These are either based on 12 questions regarding drinking behavior and the subject's report of drinking-related health problems or on eyes closed resting event-related potential (ERP) measures to quantify brain activity, possibly defining a phenotype closer to a biological pathway.

To localize susceptibility regions for alcoholism we first separately construct one phenotype for drinking behavior and two for ERP measures, pattern (P) and magnitude (M), taking clustering within families into account. Genetic information is not used in this step. Previous studies of ERP focused on the amplitudes of measurements. However, the pattern of measures can be more important than the magnitude (e.g., LDL:HDL ratio, CD4:CD8 ratio of lymphocytes). For ERP we considered both the pattern of an individual's ERP measurements and the magnitude (amplitude). Secondly, we performed a genome-wide linkage search with microsatellite markers and an association search with single-nucleotide polymorphisms (SNPs). Then we compared the results yielded by the different phenotypes and searches.

## Methods

### General phenotype construction

Behavioral questions (BQ) and ERP measures are multidimensional. We employed a reduction to a single score each while conserving as much information in the whole set as possible. For inheritance patterns the pedigree structure must be considered. Before utilizing these scores in subsequent linkage analysis, we evaluated if a score discriminates affected and unaffected siblings within a sibship. Therefore the mean difference Δ_i _of the score for "affected" and "pure unaffected" sibs of family i was calculated. Under the null hypothesis of no discrimination by the score the distribution of Δ_i _must be located around E(Δ) = 0. We performed a sign rank test and calculated a standardized effect size (δ = /SD) for each phenotype considered. As disease status we used the DSM-IV classification of alcohol dependence (in GAW14 coded as ALDX2) [1]. When applicable we adjusted for covariates (age, gender, ethnicity). Phenotype scores were calculated for all available individuals in the Collaborative Study on the Genetics of Alcoholism (COGA) dataset, even when score coefficients were estimated from a particular subset.

### ERP score

**Figure 1:**
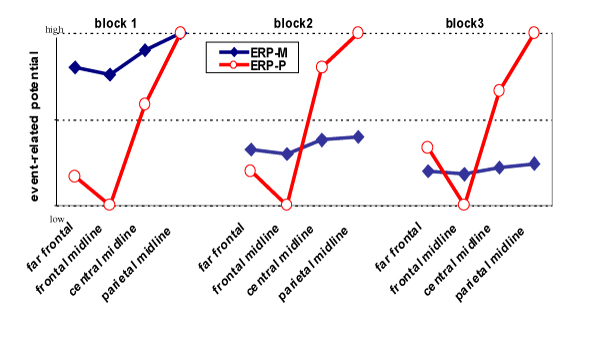
**An example of magnitude and pattern of multidimensional ERP measures for combining to univariate phenotypes**. ERP measures are projected as profiles. Measures within a block (block 1: late time window – 3–7 Hz wave band, block 2: late time window – 1–2.5 Hz wave band, block 3: early time window – 3–7 Hz wave band) are connected. Within each block measures are taken at different locations on the skull. The dotted line represents the mean of all measures. Blue, ERP-M taking into account structure + magnitude: measures of block 1 are much larger than eothers (block 2 and 3), independent from location on the skull. Red, ERP-P taking into account structure: measures of the central and parietal midline channel are larger than those of the frontal channels, this pattern is congruent across blocks.

Prior to score definition, we analyzed the impact of covariates on pure ERP measures, independent of wave band, time window, and skull location, with a generalized additive model (GAM) [2]. We considered the GAM residuals as adjusted measurements. With the GAM model, age can be incorporated as a continuous variable without assuming linear dependency or without categorizing age. ERP measures are given in three blocks defined by time and wave band (block 1: late – 3–7 Hz, block 2: late – 1–2.5 Hz, block 3: early – 3–7 Hz). Skull location was a factor within each block. As illustrated in Figure 1 (ERP-P), the magnitude and the block of a single measurement contributes much more to the variance of all 12 measures than its location on the skull. To remove differences in the order of the magnitude of the observations we standardized the ERP measures of an individual within a block using Huber's 1-step M-estimate to obtain a robust estimate of the within-block mean [3]. As illustrated in Figure 1 (ERP-P), the location on the skull contributes more to the variance than its magnitude for standardized measures. This pattern is congruent across blocks. Next we performed principal component analyses (PCA) within all sibships, for both types of ERP measures. The score of the first factor of each PCA is used afterwards as the ERP-P phenotype based on standardized measures or as the ERP-M phenotype based on nonstandardized measures, respectively. The ability of both phenotypes to discriminate affected and unaffected siblings within a sibship was evaluated as described before.

### Behavioral score

To construct a univariate score for behavior questions we applied a logistic regression model to discriminate best between "affected" and "unaffected with some symptoms" conditioning on whole families. "Pure unaffected" were not considered, because they did not show positive answers on any behavior item. Item 5 "Spent most time for drinking" was dichotomized to "<1 month" and "≥1 month". All 12 behavior items and age were included as explanatory variables. Gender did not show a significant impact. All family data were used. Scores for "pure unaffected" were also calculated according to the estimated coefficients, which is equivalent to the age effect only. To reach approximate normality as needed for further analysis the score was transformed by addition of a constant and logarithmic transformation (denoted by *lnBQ*).

### Genome-wide searches

The nonparametric linkage genome scan with microsatellites was carried out for phenotypes *lnBQ*, *ERP-P*, and *ERP-M *and bivariate phenotypes using variance component analysis implemented in SOLAR [4]. We performed multipoint analyses for autosomal and two-point analyses for the X chromosome (due to SOLAR limitations) without covariates. LOD scores were computed in 5-cM intervals. For stronger signals (LOD score >1) we rescanned in 1-cM intervals. We also carried out a second pass conditional on the QTL of the highest LOD score detected in the first pass. Because the residual kurtosis of all variables was within the normal range, standard nominal *p*-values were used [5]. Our primary goal was to compare linkage results for these phenotypes at a screening level of LOD > 1.

The association genome scan for autosomes, but not for the X chromosome, was performed using SNPs (cleaned data) of the Affymetrix and Illumina chips. Each SNP was tested for association using the FBAT program [6] with the defined uni- and bivariate phenotypes. Multigenerational pedigrees were split into nuclear families for which FBAT accounts for an arbitrary number of offspring and missing parents. We tested the hypothesis of no association allowing for linkage in the univariate case, whereas in the bivariate case we tested the hypothesis of no association and no linkage due to program limitations. To correct for correlations among sib genotypes and among nuclear families of the same extended pedigree we used an empirical variance covariance estimator when allowing for linkage [7]. Motivated by the family-based association test theory *lnBQ *was centred to yield mean zero. The level of significance was set to α = 5%. In addition to raw *p*-values, an adjustment by the false discovery rate method of Benjamini and Hochberg was considered [8].

All calculations and data handling were done with R or SAS^® ^8.6.

## Results

### Phenotypes based on ERP

All original ERP measures were significantly affected by sex (men show in the mean 0.25 lower values than women, *p *< 0.0001) and non-linearly by age (*p *< 0.0001). Age is estimated as u-shaped with a minimum around 45 years. The largest effect by ethnicity (*p *< 0.0001) showed the contrast of Black non-Hispanic (lowest mean) compared with American Indian (in the mean 0.62 higher), but other populations also differed from each other. Almost half (49.6%) of the variability of the adjusted, nonstandardized ERPs is explained by the first principal component. The score coefficients (see Table 1) are almost the same (range 0.08–0.14). Hence ERP-M is well approximated by the mean of all 12 ERPs and might be interpreted as overall brain activity. The first principal component for adjusted and standardized measures explains only 30% of the overall variability and here the score coefficients vary (Table 1). This contrasts with the ERPs from the forehead with those located central and parietal on the skull. Hence ERP-P might be interpreted as a within-brain activity contrast.

**Figure 2:**
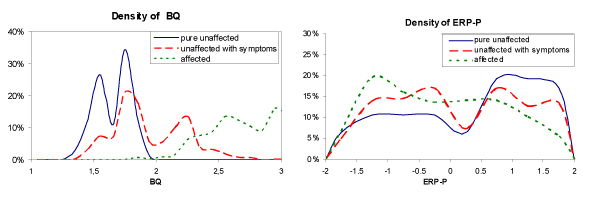
**Density (kernel smoothed) of lnBQ and ERP-P scores of sibs**.

Visual inspection of the phenotype density functions (Figure 2) shows that differences between affected and unaffected individuals are small compared with the observed range. The effect sizes for the scores defined above were δ = 0.47 for ERP-M and δ = 0.30 for ERP-P. Assuming normality the probability for correct group assignment based on the score is 0.59 and 0.56, respectively (0.5 is the lower bound) [9]. For both scores we could achieve a significant difference comparing affected and pure unaffected siblings, nested within their sibships (*p*_ERP-M _= 0.0002, *p*_ERP-P _= 0.017).

### Phenotype based on behavioral questions BQ

The applied logistic model yielded a pseudo-R^2 ^of 0.527 (max possible = 0.602). Score coefficients are given in Table 1. Item 1, "persistent desire to stop drinking" (OR = 27.2, 95% CI: 11.5–63.9), and item 8, "blackouts" (OR = 11.2, 95% CI: 5.1–24.5), achieved the highest coefficients. When comparing the groups of "affected" and "pure unaffected" by the derived *BQ *score an effect size of δ = 2.13 could be achieved. This may be considered equivalent to a group assignment being ~85% correct (Figure 2) [9]. This *BQ *score was found to be u-shaped distributed over all ALDX2 groups, unimodal and skewed for 'affected' and for 'unaffected with symptoms'. Because 'pure unaffected' answered with 'no' for all 12 items, they achieved low scores due to the age coefficient only. The age coefficient was negative, which indicates that the score derived from an identical answer profile is less in older than in younger individuals. The *BQ *score has than been transformed to reduce skewing (*lnBQ*) and used later.

### Genome-wide linkage searches with microsatellites

**Figure 3:**
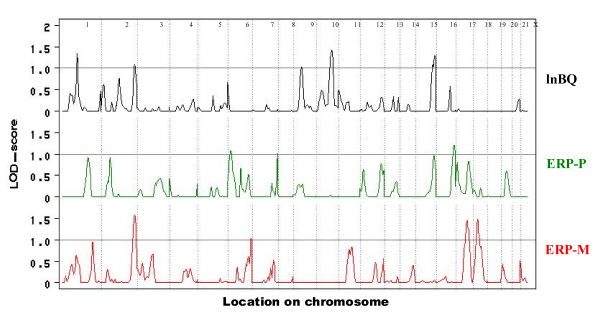
**LOD score results for lnBQ, ERP-P and ERP-M (first pass)**. For the autosomes the x-axis shows map location in cM and for the X chromosome the markers numbered 1–13.

For the *lnBQ *five loci achieved a LOD > 1 (on chromosomes 1, 2, 9, 10, 15; all using the 1-cM density map), with the highest LOD score on chromosome 10 at 114 cM between D10S670 and D10S544 (max LOD = 1.4). For *ERP-P *three loci achieved a LOD > 1 (on chromosomes 6, 7, 16; all using the 1-cM density map), with the highest LOD score on chromosome 16 at 130 cM between D16S750 and D16S539 (max LOD = 1.2). For *ERP-M *four loci achieved a LOD > 1 (on chromosomes 2, 6, 17, 18; all using the 1-cM density map), with the highest LOD score on chromosome 2 at 243 cM between D2S434 and D2S1323 (max LOD = 1.6). In second passes for all three phenotypes, conditional on the most significant QTL, no further evidence for linkage (LOD > 1) was found. The scans for the three phenotypes result in linkage signals at different locations across the genome, except for those on chromosomes 2 for *lnBQ *and *ERP-M *and 15 for *lnBQ *and *ERP-P *(see Figure 3). Bivariate scans confirmed these shared findings.

### Genome-wide association searches with SNPs

**Figure 4:**
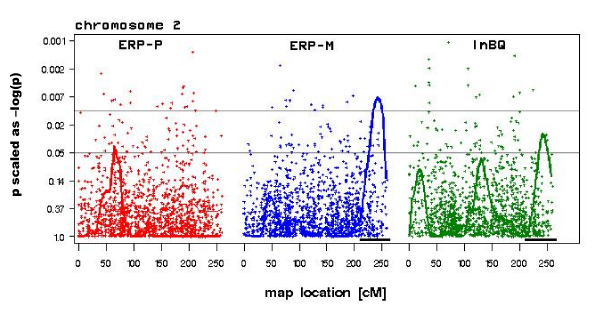
**Comparison of nonparametric linkage and association test results for lnBQ, ERP-P and ERP-M on chromosome 2**. LODs have been transformed to *p*-values by the relationship LOD = χ^2^_df = 1_/2ln(10) and plotted as a solid line. Dots represent family-based association test results.

We achieved for *lnBQ*, *ERP-M*, and *ERP-P *745, 807, and 734 nominally significant results out of 15264–15268 SNPs tested for association. This corresponds to 4.9%, 5.3%, and 4.8%, respectively, of all SNPs and hence is expected under the null hypothesis of no association for any marker. These significant SNPs are spread over the whole genome. Thus, most of the significant results should be false positives and causal associations seem to be rare. After adjusting the *p*-values by the false discovery rate method none of the SNPs remains significant. A total of 2,175 SNPs are located within 11 linkage regions (± 30 cM around a LOD peak >1.0). Within these regions 0.0% to 7.11% are significantly (at the 5% level) associated with *lnBQ, ERP-P*, or *ERP-M*, respectively. Figure 4 shows as an example the association and linkage results on chromosome 2.

## Conclusion

For the identification of susceptibility loci in complex diseases, the choice of the target phenotype, the marker set, and the analysis strategy are important issues. For alcoholism one might expect an applicable impact of the social surrounding. As biophysiological measures we derived two different phenotypes, *ERP-M *and *ERP-P*, from ERP measures. The poor ability to discriminate between affected and unaffected individuals may result from a small number of informative discordant sibships (*n *= 49) in the sample, weak classification of affection status by DSM-IV, or the properties of ERP-M and ERP-P as phenotypes, which were chosen specifically to be different from each other. We could identify 11 candidate regions for linkage on 10 chromosomes using all three phenotypes. Only one region (chromosome 2: 212–273 cM) was found twice, for *lnBQ *and *ERP-M. *The region on chromosome 15 (117 cM to end) was identified clearly by *lnBQ *and showed a maximal LOD of 0.95 related to *ERP-P*. The three phenotypes differ in their information, so that heterogeneous findings are expected. *ERP-M *and *ERP-P*, different scores calculated on the same set of measurements, can be seen as overall brain activity (ERP-M) and within-brain activity contrast (ERP-P). Here the usability of these scores cannot be discussed regarding biophysiology, but only regarding results.

Some linkage peaks are replications from previous studies. For example, 3 of the 4 linkage regions identified for *lnBQ *have been reported before. Analyzing the COGA dataset, Reich et al. [10] reported the same region on chromosome 1 for alcoholism as a qualitative phenotype, and Nurnberger et al. [11] found linkage on the same regions of chromosomes 1 and 2 to the phenotype alcoholism or depression. Interestingly, the region on chromosome 9 was identified before in the Framingham Heart Study [12,13].

Genome-wide association tests of SNPs (*p*-value ≤ 0.05) within these regions were checked to determine if they were on a gene potentially relevant for alcoholism. On chromosome 10 we found a SNP lying on SORCS3 that showed a significant association with *lnBQ *(rs1361800, *p *= 0.027). SORCS3 potentially encodes for a neurotensin receptor.

We also checked the ± 30 cM regions around every LOD-score peak for known or possible candidate genes related to alcoholism [14,15]. The gene of a dopamine responsive protein (LOC220869) is located in the chromosome 9 area for *lnBQ*. We found HTR7 encoding serotonin receptor 7 and COMTD1 in the chromosome 10 area for *lnBQ*. The gene of μ-opiod receptor (OPRM1) is located in the chromosome 6 area for *ERP-M*. Finally the gene SLC6A4 (serotonin transporter) is located about 30 cM away from the LOD peak on chromosome 17 for *ERP-P*. It should be noted that here we did not account for sex differences in linkage maps. Furthermore, we noticed differences between SNP and microsatellite marker maps up to 40 cM. Both can generate some false location comparisons (Figure 4).

## Abbreviations

BQ: Behavioral questions

COGA: Collaborative Study on the Genetics of Alcoholism

ERP-M: Event-related potential magnitude

ERP-P: Event-related potential pattern

GAM: Generalized additive model

GAW14: Genetic Analysis Workshop 14

SNP: Single-nucleotide polymorphism

## Authors' contributions

The authors discussed research questions, analysis, results and next steps as a team. Special tasks were phenotype definition (AR, K Köhler, NJ), linkage analysis (NJ, BK), association analysis (K Köhler, BK), candidate genes (K Korb), and drafting the article (AR, HB). All authors gave their final approval of this manuscript.

## Figures and Tables

**Table 1:** Score coefficients for ERP-P, ERP-M and BQ

ERP:	ERP-P	ERP-M	Behavior questions (BQ)	
	
HCM	Coefficients		Item	Coefficient
	
ttth1	-0.389	0.121	Age [years]	-0.043
ttth2	-0.222	0.139	1. Persistent desire to stop drinking	3.302
ttth3	0.043	0.132	2. Morning drinking	0.581
ttth4	0.000	0.128	3. Craving	1.487
ttdt1	-0.326	0.085	4. Ever binge drink	0.779
ttdt2	-0.228	0.102	5. Spent most time for drinking	1.670
ttdt3	0.016	0.112	6. Narrowing of drinking repertoire	0.696
ttdt4	0.000	0.101	7. Gave up activities to drink	2.020
ntth1	-0.352	0.107	8. Blackouts	2.412
ntth2	-0.211	0.133	9. Withdrawl SXs	0.247
ntth3	0.039	0.132	10. Physical health problems	0.134
ntth4	0.000	0.116	11. Emotional/psychological problems	0.694
